# A new consensus document on electrocardiographic interpretation in athletes: does it help to prevent sudden cardiac death in athletes?

**DOI:** 10.1007/s12471-018-1076-6

**Published:** 2018-02-01

**Authors:** N. M. Panhuyzen-Goedkoop, H. T. Jørstad, J. L. R. M. Smeets

**Affiliations:** 1 Academical Medical Centre Amsterdam, Amsterdam, The Netherlands; 2 Radboud University Medical Centre, Radboudumc, Nijmegen, The Netherlands

**Keywords:** Athlete, ECG, Sudden cardiac death, Pre-participation screening, Prevention

## Abstract

Sudden cardiac arrest or death (SCA/SCD) in athletes has a low event rate. Pre-participation or eligibility screening is a widely accepted method of primary prevention of SCA/SCD in athletes. Most European countries and international sports governing bodies perform ECG-inclusive screening. However, implementation of a resting 12-lead ECG in pre-participation or eligibility cardiac screening is still a topic of debate. Recently, the ‘International recommendations for electrocardiographic interpretation in athletes’ was published in three leading international medical journals. These international ECG criteria are based on studies with detailed information on resting 12-lead ECG of Caucasian and Afro-Caribbean athletes or on consensus in case evidence was lacking. Normal, borderline and abnormal ECG findings in young athletes (age 12–35 years) are clearly described and illustrated to assist the screening physician in interpreting ECGs of athletes correctly.

In this ‘point of view paper’ we will discuss whether these new ECG criteria actually help prevent SCA/SCD in athletes.

## Introduction

Sudden cardiac arrest and/or death (SCA/SCD) in athletes is a very tragic event that attracts a lot of media attention. The key question conventionally raised after such an event resulting from lethal ventricular arrhythmia, i. e. ventricular tachycardia/fibrillation (VT/VF), is: could this have been prevented? SCD in competitive athletes aged 35 years and younger (young athletes) is rare (0.6–2.85/100,000 annually). The incidence is considerably lower than in the overall population (3–10.7/100,000 annually) and significantly lower than the incidence of VT/VF in Europe (84.0/100,000 annually) [1–7]. Most inherited and congenital cardiovascular diseases (CVD) in athletes at risk of VT/VF can be identified during life [2, 3]. To date, primary prevention with pre-participation or eligibility cardiac screening is a widely accepted method to reduce SCA/SCD in athletes [8–10]. If pre-participation or eligibility cardiac screening fails to identify athletes at risk, secondary prevention with bystander resuscitation, including defibrillation with automatic external defibrillator (AED), is essential to save an athlete’s life [11]. However, an AED is no adequate replacement for pre-participation or eligibility cardiac screening [11]. How we need to screen athletes for conditions predisposing to VT/VF optimally is a topic of debate.

In this manuscript we discuss the ‘International recommendations for electrocardiographic interpretation in athletes’, questioning if these new ECG criteria help preventing SCA/SCD in athletes [12–14].

## Pre-participation cardiac screening

The purpose of pre-participation or eligibility cardiac screening in athletes is identifying CVD at risk of VT/VF and reducing SCA/SCD by disease management [8–10]. Pre-participation or eligibility cardiac screening consists of personal and family history taking and physical examination [1, 8–10, 15]. Pre-participation or eligibility cardiac screening performed by most European countries and international sports governing bodies include a 12-lead resting ECG [1, 8, 16]. In the Netherlands, pre-participation or eligibility cardiac screening is performed by sports physicians according to the ‘Lausanne protocol’ [15]. If pre-participation or eligibility cardiac screening results raise suspicion of a CVD at risk of VT/VF additional cardiac evaluation is recommended before clearing the athlete [8, 9, 17]. To adequately perform pre-participation or eligibility cardiac screening, the screening physician needs training and skills in physiology, ECG interpretation, CVD at risk of VT/VF, and CVD management in athletes [10, 18].

## Athlete ECG

In 2010, the European Society of Cardiology (ESC) for the first time classified athlete ECGs in training-related and training-unrelated or pathologic ECG findings [16]. Training-related ECG findings induced by vagotonia and volume and/or pressure overload of the cardiac cavities are an expression of athlete’s physiologic cardiac adaptation or remodelling [16, 19, 20]. The ESC 2010 criteria were principally based on a large Italian registry in almost exclusive Caucasian young athletes [2, 16]. Left atrial enlargement, cardiac axis deviation and criteria of right ventricular hypertrophy (RVH) were classified as pathologic ECG changes [2, 16]. However, the typical ECG changes of left ventricular hypertrophy (LVH) and ST-T changes in Afro-Caribbean ethnicity were not mentioned and therefore regarded as abnormal. This resulted in a high false positive rate of ECG-inclusive screening (FPR 8.8–26.5%) [20–22].

The Seattle 2013 criteria were based on ECG data in both Caucasian and Afro-Caribbean athletes [23, 24]. ‘Convex ST-segment elevation combined with T‑wave inversion in leads V1-4 in Afro-Caribbean athletes’ was classified as training-related [24]. However, left atrial enlargement, cardiac axis deviation and RVH were still classified as abnormal [25].

The so-called ‘borderline ECG findings’ were introduced by Sheikh et al. because the issue of the resemblance of an athlete’s ECG and pathology had not been solved [20]. The Refined criteria regarded left atrial and right atrial enlargement, cardiac axis deviation, RVH and T‑wave inversion in leads V1-4 in Afro-Caribbean athletes normal findings if considered in isolation, but abnormal if two or more patterns were present [20]. Using the Refined criteria, a lower FPR was observed in a cohort of predominantly young (≤35 years) male Caucasian (FPR 6.1%) and Afro-Caribbean (FPR 15.8%) athletes (Table 1; [20]). A lower FPR using the Refined criteria was also observed in an Arabic study in young male athletes (FPR Caucasians 2.5%, Afro-Caribbeans 3.1%) and in adolescent soccer players (FPR Caucasians 2.1%, Afro-Caribbeans 9.2%) (Table 1; [21, 22]). Although ECG screening demonstrated different FPR results in non-comparable cohort studies the Refined criteria were a major step forward in evidence-based ECG interpretation in athletes.

**Table 1:** Comparison of three different sets of ECG criteria in ECG-inclusive screening in three cohorts of young athletes

		ESC 2010 criteria		Seattle criteria		Refined criteria	
Reference	Cohort	Caucasian	Afro/Caribbean	Caucasian	Afro/Caribbean	Caucasian	Afro/Caribbean
		FPR (%)	FPR (%)	FPR (%)	FPR (%)	FPR (%)	FPR (%)
Sheikh [20]	Caucasian 4,297, Afro/Caribbean 1,208; male 94.2%; 2000–2012	26.5	59.9	7.9	20.7	6.1	15.8
Riding [21]	Caucasian 367, Afro/Caribbean 748; male 100%; 2010–2014	12.6	15.5	3.9	5.3	2.5	3.1
Malhotra [22]	Caucasian 9,262, Afro-Caribbean 894; adolescent soccer players	18.6	29.1	8.5	15.8	2.1	9.2

*ESC *European Society of Cardiology*, FPR *false positive rate

Research, in particular that of Sharma et al. (London, United Kingdom), conducted to describe in detail ECGs of athletes of different ethnicity, gender and intensity of sports participation has been of great value for the consensus of the international ECG criteria [12–14, 19, 20, 26, 27]. This third document, endorsed by several international (European Society of Cardiology, American Heart Association, American College of Cardiology) and national cardiac societies, sports medicine societies and sports governing bodies, is an updated practical consensus for the screening physician to interpret and recognise physiologic and pathologic ECG findings in athletes [12–14].

## Normal, borderline and abnormal ECG findings in athletes

The international consensus ECG criteria describe and illustrate each separate ECG finding at rest in young athletes [12–14]. The ECG findings are classified as normal, borderline and abnormal.

**Fig. 1:**
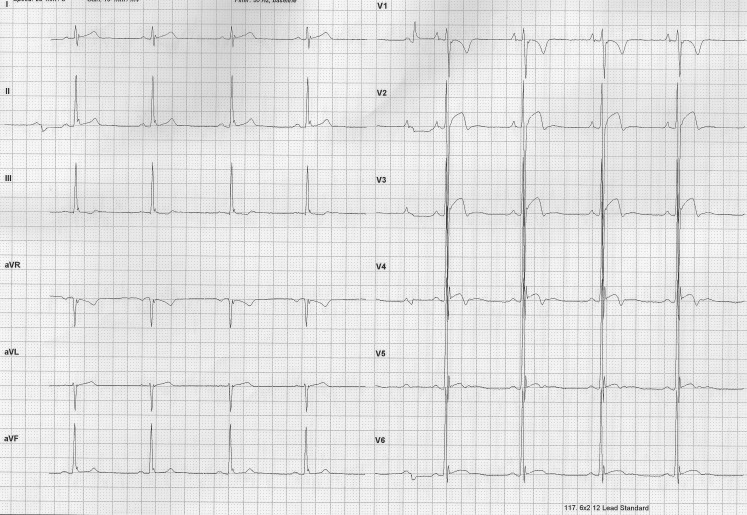
ECG of an asymptomatic 21-year-old male Afro-Caribbean elite soccer player with a negative family history. Legend explanation: PR-interval <120 ms and <2.5 mV; J‑elevation with convex ST-elevation and T‑wave inversion in leads V2-4; increased QRS voltage criteria for LVH. In conclusion: normal ECG. Eligible to play. *LVH* left ventricular hypertrophy, *ECG* electrocardiogram. (Copyright 1999–2001 Jaeger bv)

*Normal or training-related ECG findings* are induced by long-term sports participation on a regular basis for at least four hours per week [12–14]. As is mentioned above, these ECG findings at rest reflect increased vagotonia and cardiac remodelling in athletes (Table 2; Fig. 1). Such ECG findings warrant no further cardiac evaluation and the athlete is eligible to play.

**Table 2:** International ECG criteria: normal and borderline ECG findings in athletes 35 years and younger [12–14]

Cardiac adaptation	Normal ECG finding	Borderline ECG finding
Vagotonia	Sinus bradycardia or arrhythmia	
	Ectopic atrial or junctional rhythm	
	1st degree AV block	
	Wenckebach AV block	
Changes of cavity size and wall thickness (cardiac remodelling)	Incomplete RBBB	Complete RBBB
	Increased QRS voltage for LVH or RVH	Left atrial enlargement
	Early repolarisation/ST-segment elevation	Right atrial enlargement
	ST-segment elevation followed by T‑wave inversion leads V1-4 in Afro-Caribbean athletes	Left axis deviation
	T-wave inversion leads V1-3 age 16 years and younger	Right axis deviation

*AV* atrioventricular, *ECG* electrocardiogram, *RBBB* right bundle branch block, *LVH* left ventricular hypertrophy, *RVH* right ventricular hypertrophy

*Borderline ECG findings* are left atrial and right atrial enlargement, electrical axis deviation and complete right bundle branch block (RBBB). When found in isolation in asymptomatic athletes with a negative family history of inherited CVD or SCA/SCD these findings are classified as normal and need no further cardiac evaluation (Table 2; [12–14]). However, when two or more borderline findings are present and/or the athlete is symptomatic and/or has a positive family history, these borderline ECG changes are classified as abnormal and additional cardiac evaluation is warranted before clearing the athlete [12–14].

**Fig. 2:**
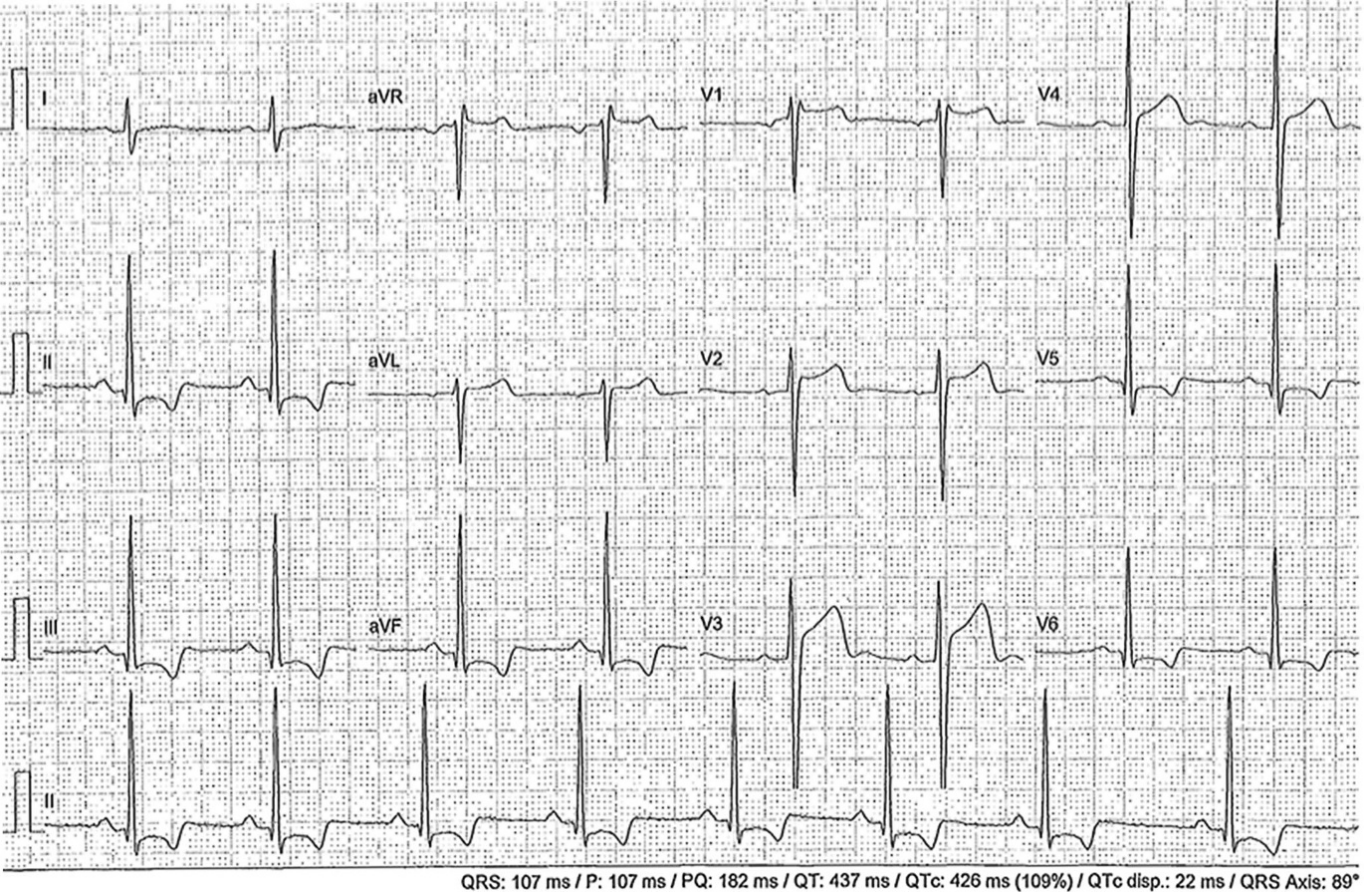
Asymptomatic 23-year-old male Caucasian elite soccer player, negative family history, 5 years ago viral myocarditis. Legend explanation: PR interval <120 ms and <2.5 mV (normal finding); ST-depression with negative T‑waves in leads II-III-aVF, V5-6, i. e. inferolateral (abnormal finding). In conclusion: abnormal ECG. Perform additional cardiac evaluation with echocardiography and magnetic resonance imaging to rule out cardiomyopathy, hypertrophic or otherwise. Not eligible to play until further notice

**Fig. 3:**
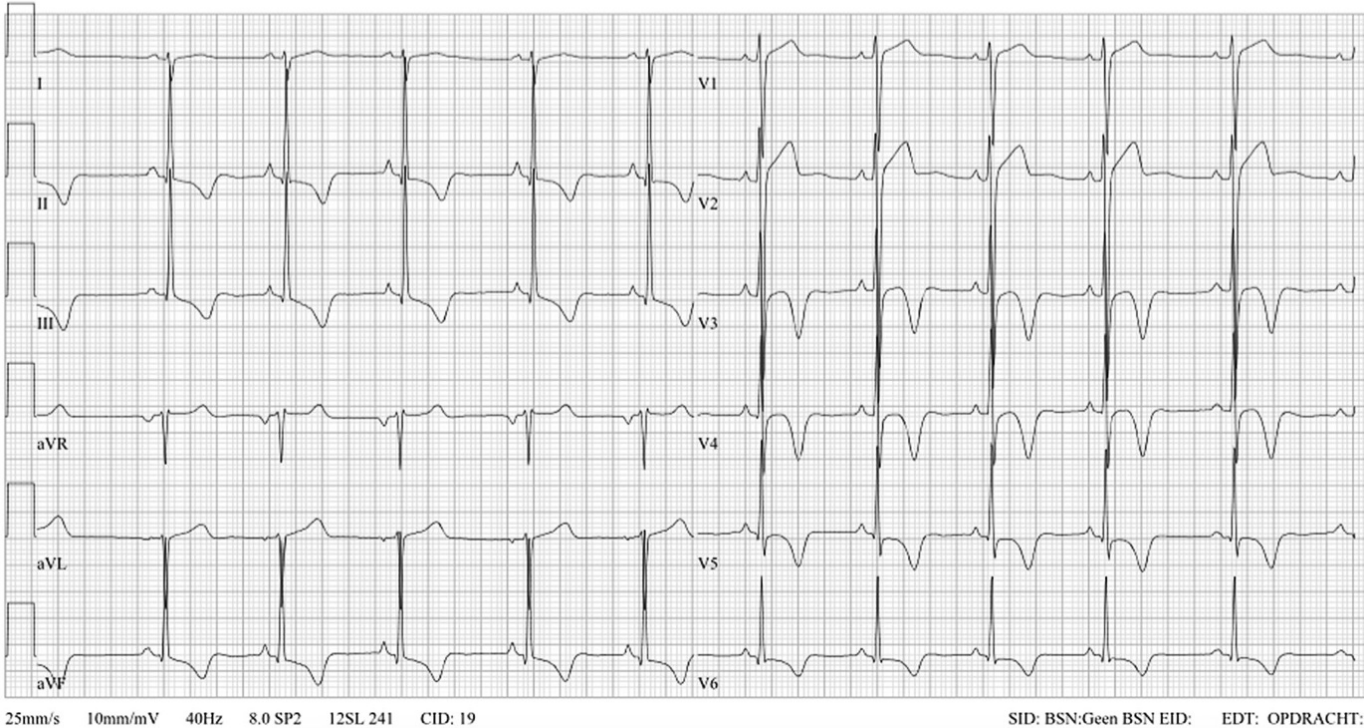
Asymptomatic 21-year-old male Caucasian elite soccer player, on army check-up his 26-year old brother was referred for cardiac evaluation because of an abnormal ECG. Legend explanation: PR interval <120 ms and <2.5 mV (normal finding); increased QRS voltage criteria for LVH (normal finding); ST-depression with deep negative T‑waves in leads II-III-aVF, V3-6, i. e. infero-antero-lateral (abnormal finding). In conclusion: abnormal ECG. Perform additional cardiac evaluation with echocardiography and MRI to rule out cardiomyopathy. Not eligible to play until further notice. *LVH* left ventricular hypertrophy, *ECG* electrocardiogram, *MRI* magnetic resonance imaging

*Abnormal ECG findings* are pathologic changes until proven otherwise. These abnormal findings reflect CVD at risk of VT/VF (i. e. inherited or congenital CVD, myocarditis, coronary disease) recommending additional cardiac evaluation before clearing the athlete to participate in sports (Figs. 2 and 3). The international ECG criteria clearly describe and illustrate the details of abnormal ECG findings suspicious of CVD at risk of VT/VF [12–14].

## Does this international consensus document help to prevent VT/VF in athletes?

The consensus-based recommendations for ECG interpretation in athletes assist the screening physician in identifying athletes at risk of VT/VF. However, false positive rate (FPR) and false negative rate (FNR) of pre-participation or eligibility cardiac screening remain a problem. On the one hand, false positive test results wrongly identify athletes at risk of VT/VF, lead to unnecessary additional cardiac evaluation at high costs and result in uncertainty about the athlete’s career [27, 28]. On the other hand, wrong eligibility decision-making in false negative test results can put athletes at risk of VT/VF [28, 29]. To reduce FPR results, the screening physician must be well-trained in interpreting ECGs of athletes and is recommended to use the most recent ECG criteria describing the lowest FPR [18, 20–22, 27, 30]. Future studies need to demonstrate that the international ECG criteria will result in a lower or equal FPR as compared with the Refined ECG criteria. However, there are several limitations inherent to ECG-inclusive pre-participation or eligibility cardiac screening, including the interpretation of an ECG at rest alone. Sometimes the ECG changes are very subtle and difficult to recognise. In myocarditis and cardiac concussion in blunt chest trauma these subtle ST-T changes are not always initially present. Furthermore, certain congenital and inherited CVDs (i. e. coronary anomaly, premature coronary syndrome, catecholaminergic polymorphic ventricular tachycardia) cannot be identified at a resting-ECG alone. Besides, abnormal ECG findings of a CVD at low risk of potential lethal cardiac events (i. e. atrial fibrillation, AV nodal re-entry tachycardia) are a confounder of the purpose of pre-participation or eligibility cardiac screening to identify athletes at risk [1, 4, 5, 10]. Finally, ECG findings in other ethnicities, such as Hispanic, Asian and mixed ethnicity, and in athletes over 35 years of age and children are not included in this consensus document.

## Conclusion

ECG-inclusive pre-participation cardiac screening to prevent SCA/SCD in athletes is implemented by most European countries and international sports governing bodies. The international ECG criteria, endorsed by international cardiac and sports medical societies and sports governing bodies, pose an updated and clear guide in interpreting ECGs of athletes to appropriately identify abnormal ECG findings at rest suspicious of CVD at risk of VT/VF. The screening physician must be trained in interpreting ECGs of athletes following the most recent ECG criteria to avoid wrong decision-making. Further studies are needed to determine if these updated international ECG criteria help to prevent SCA/SCD in athletes (12–35 years of age).
